# Colorectal surgeons’ perspectives on the efficacy of intraoperative bowel perfusion technology with a focus on indocyanine green fluorescence angiography

**DOI:** 10.1007/s00423-025-03640-9

**Published:** 2025-02-17

**Authors:** Ashokkumar Singaravelu, Philip D. Mc Entee, Patrick A. Boland, Alice Moynihan, Cathleen McCarrick, Alexander L. Vahrmeijer, Alberto Arezzo, Luigi Boni, Roel Hompes, Ronan A. Cahill

**Affiliations:** 1 UCD Centre for Precision Surgery, University College Dublin, 47 Eccles St, Dublin, Ireland; 2 Department of Surgery, Mater Misericordiae University Hospital, Dublin, Ireland; 3 Department of Surgery, Leiden University Medical Center, Leiden, The Netherlands; 4 Department of Surgical Sciences, University of Turin, Turin, Italy; 5 Department of General and Minimally Invasive Surgery, Fondazione IRCCS Ca’ Granda Ospedale Maggiore Policlinico di Milano, Milan, Italy; 6 Department of Surgery, Amsterdam University Medical Centre, Amsterdam, The Netherlands

**Keywords:** Indocyanine green fluorescence angiography, Colorectal resections, Technology, Surgeon, Perceptions, Survey

## Abstract

**Background:**

Level one evidence supports indocyanine green fluorescence angiography (ICGFA) use reducing anastomotic leak rates in colorectal surgery. We surveyed surgeons exploring perceptions and factors affecting its use in daily practice and adoption as routine standard of care.

**Methods:**

Validated electronic survey distributed via the Irish Association of Coloproctology, Royal College of Surgeons in Ireland, European Society of Surgical Oncology, European Association for Endoscopic Surgery, Milan Colorectal Congress and social media.

**Results:**

200 colorectal surgeons (143 consultants) responded. 147 (73.5%) surgeons already use ICGFA, with 90 (61.2%) using it routinely and 69 (46.9%) having a concomitant research interest. Strong clinical evidence base (83.5%) and protocol standardisation (78%) were overall rated most important for bowel perfusion technology with a majority of surgeons rating lack of standardisation and inter-user variability as challenges (similar between consultants and non-consultants). Lack of training and staff, reliability concerns and data security were perceived as significant barriers by selective users compared to non-users, and cost and operating time were perceived as significant barriers by selective users compared to routine users. Most surgeons (41.5%) ideated a number needed to treat (NNT) between 20 and 40 acceptable to advocate routine ICGFA use with 28% requiring a NNT < 20. Most surgeons (38.5%) indicate a per case cost savings of €250–500 supports routine use with 17% advocating it > €750.

**Conclusions:**

With now a strong evidence base regarding clinical benefit, the survey respondents articulate remaining challenges for ICGFA as standard of care. Levels of expected benefit are largely in keeping with its reported performance.

**Supplementary Information:**

The online version contains supplementary material available at 10.1007/s00423-025-03640-9.

## Introduction

Anastomotic leak (AL) is a severe complication of colorectal surgery and is associated with significant morbidity, mortality and economic burden [1]. AL rates are reported as high as 27% and several risk factors have been identified including tumour height from the anal verge, obesity, smoking, alcohol consumption, and preoperative radiotherapy [2–4]. Hypoperfusion resulting in poor anastomotic healing is a known mechanism for AL [5]. While other factors may cause AL even in the presence of sufficient perfusion, at present ensuring sufficient perfusion seems to be the single greatest factor which the surgeon may influence as part of a sound surgical technique.

Various intraoperative bowel perfusion assessment technologies have been described in the literature to support surgeon judgement alone including indocyanine green fluorescence angiography (ICGFA), diffuse reflectance spectroscopy (DRS), hyperspectral imaging (HSI), and laser speckle contrast imaging (LSCI) [6]. Among these technologies, ICGFA is the most broadly adopted [6], with all major surgical camera manufacturers now offering near-infrared imaging capabilities. A recent meta-analysis of randomised controlled trials (RCTs) has shown that the use of ICGFA is associated with a significantly lower rate of AL in rectal cancer surgery [7–9]. Given that it may so be reaching the evidential threshold to be proposed as the standard of care, it would seem reasonable now to consider the perceptions of users and, perhaps especially, non-users regarding intraoperative bowel perfusion assessment technology in order to identify particularly remaining challenges and obstacles to use.

To give this context, we surveyed colorectal surgeons broadly with the aim of assessing current usage of bowel perfusion technology with a focus on ICGFA, exploring factors affecting adoption and use, and assessing the level of clinical and economic benefits needed in considering routine application of ICGFA during colorectal resections.

## Methods

The Human Research Ethics Committee – Sciences (HREC-LS) of University College Dublin approved this work as a low-risk study (LS-LR-23-263-Singaravelu-Cahill). For the survey, a standardised questionnaire was designed using the Google Forms platform- the first draft of questions (created by AS) was reviewed and validated iteratively three times by a panel of four surgeons with extensive experience in the field of intraoperative perfusion assessment technology (PMcE, PAB, AM, RC, see supplementary file) to ensure that questions and response options were clear and aligned with the objectives. We assessed whether surgeons would prefer a number needed to treat (NNT) lower than the calculated value (NNT = 22 [7]) or if they would find a higher NNT acceptable (NNT 20–40, or 40–60, or > 60) with similar extrapolation of an estimated cost savings range from the literature [10]. An electronic link to the final version was shared accessible networks including the Irish Association of Coloproctology and the Royal College of Surgeons in Ireland association as well as via the European Society of Surgical Oncology, European Association for Endoscopic Surgery, and the Milan Colorectal Congress which all were holding their main annual congresses during the study period. Invitations were also more broadly sent via social media including Twitter (X) and Linked-In. Responses were collected between 27th February 2024 and 24th July 2024.

### Statistical analysis

Likert scale results (as ordinal data) were scrutinised for statistical trends by Wilcoxon rank sum (Mann-Whitney U) including search for any important differences (*p* < 0.05) in perspectives by professional standing (i.e. consultants vs. non-consultants), geographic jurisdictions and gender. Kruskal-Wallis test was used to compare perceptions among surgeons who routinely use ICGFA, those who selectively or rarely use ICGFA, and those who do not use ICGFA at all. If there was a significant difference between groups, pairwise comparisons using Wilcoxon rank sum test (p-value adjustment method, Benjamini-Hochberg adjustment) were performed. Stacked bar charts were created for Likert scale data using the “ggplot2”, “viridis”, “scales”, “ggthemes”, and “dplyr” packages in RStudio 2023.12.0. Pie charts were created using “ggplot2”, “viridis”, “scales”, “gridExtra”, and “grid” packages [11–17].

## Results

200 colorectal surgeons (143 consultants, 33 registrars, 9 senior house officers, 15 interns) completed the survey with 93% being based in Europe (57 in Italy, 44 in Ireland, 10 in Greece, 8 in the Netherlands, 7 in Germany and 6 each in the United Kingdom and Turkey, see supplementary Table 1). 156 surgeons were male and 42 were female with 2 preferring not to say.

### Current use of colonic perfusion assessment technology

174 (87%) believe bowel perfusion technologies positively impact AL whereas 24 (13%) believe none of the listed perfusion technologies have any impact. 154 (77% overall) respondents believed that ICGFA has the greatest impact in reducing the AL rate. 14 (7%) participants favoured HSI, five (2.5%) LSCI and three (1.5%) DRS. 148 (74%) utilise intraoperative bowel perfusion assessment technology, with 147 (73.5%) using ICGFA (three use ICGFA, HSI, one each uses DRS, ICGFA, HSI, and LSCI, ICGFA, DRS, and LSCI, ICGFA and HSI and 1 uses DRS). For the question ‘how often has intraoperative perfusion assessment technology influenced your surgical decision-making?’, 57 (38.5%) surgeons reported “often” or “very often”, 55 (37.2%) reported “occasionally”, and 36 (24.3%) reported “rarely” or “never”. For the question ‘how confident are you in the accuracy and reliability of the bowel perfusion assessment technology you use?’, 99 (66.9%) reported “confident” or “very confident”, 29 (19.6%) reported “mildly confident”, and 20 (13.5%) reported “slightly confident” or “not confident”.

Focussing on ICGFA, 90 surgeons (61.2%) use this technology routinely in all patients undergoing colorectal resection (Table 1). Of the 45 (30.6%) using it selectively, twenty-eight (62.2%) use it in patients with high-risk comorbidities (e.g., obesity, smoking, etc.) and seventeen (37.8%) surgeons use it selectively in complex surgical procedures. Twelve (8.2%) reported using ICGFA rarely. Of those who use a bowel perfusion technology, seventy surgeons (47.3%) report research interest in this field with most (68.6%) using it routinely and forty-nine being involved in clinical trials with patients only. Five surgeons were involved in preclinical research only, thirteen had engaged with both preclinical and clinical studies and four clinical trialists also had performed laboratory research. Seventy-eight surgeons have not done any research in this field.

**Table 1:** Use of intraoperative colonic perfusion assessment technology

	Consultant (*n* = 143)	Non-consultants (*n* = 57)
Bowel perfusion technology user	**109**	**39**
Male: Female: Prefer not to say	118: 25: 0	38: 17: 2
ICGFA users Routinely High-risk cases Rarely	108 69 32 7	39 21 13 5
HSI	4	0
DRS	2	0
LSCI	2	0

Abbreviation: ICGFA, indocyanine green fluorescence angiography; DRS, diffuse reflectance spectroscopy; HSI, hyperspectral imaging; LSCI, laser speckle contrast imaging

### Perceived clinical impact

136 (68%) surgeons reported intraoperative colonic perfusion assessment technology should be used routinely in all patients. Thirty-six (18%) reported that it should be used in high-risk patients only. The remaining 28 surgeons (14%) were not sure.

**Fig. 1:**
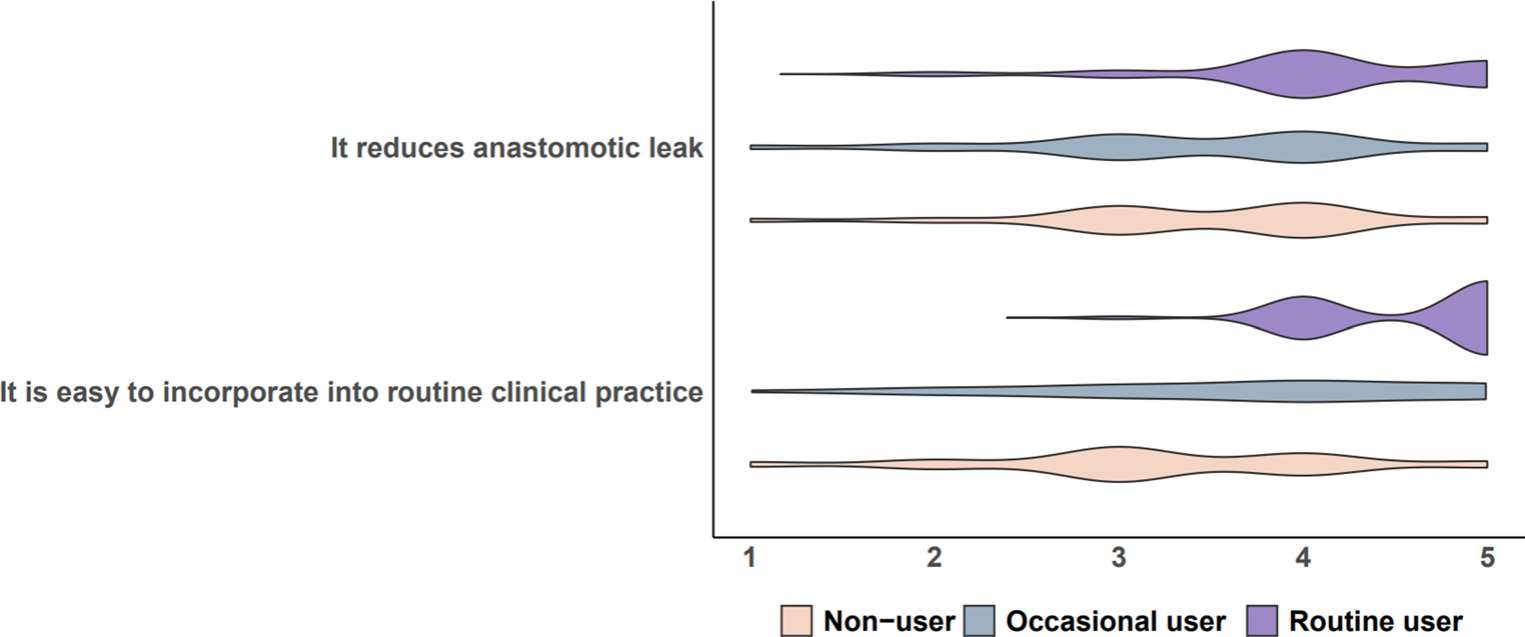
Violin plot showing the distribution of surgeons’ opinions on each statement. Responses were registered on a five-point Likert scale ranging from 1 (strongly disagree), 2 (disagree), 3 (neutral), 4 (agree), to 5 (strongly agree). ns, non-significant differences in mean scores

Overall, for the statement ‘it reduces anastomotic leaks’, 39 (19.5%) strongly agreed, 96 (48%) agreed, 47 (23.5%) neither agreed nor disagreed, 14 (7%) disagreed, and 4 (2%) strongly disagreed. Routine ICGFA users agreed statistically more with this statement then occasional ICGFA users (*p* = 1.1 × 10^− 5^) and non-users (*p* = 7.8 × 10^− 6^) (Fig. 1; Tables 2 and 3). There was neither statistical difference in responses between consultants and non-consultants (*p* = 0.14), nor between groups by the locations of the two largest geographical sites of contributions (i.e. Irish versus non-Irish surgeons (*p* = 0.144), Italian versus non-Italian (*p* = 0.695)). There was also no difference between males and females (*p* = 0.46).

**Table 2:** Perceptions of surgeons who routinely use ICGFA, those who selectively and rarely use ICGFA, and those who do not use ICGFA at all regarding agreement with two statements

	Routine ICGFA users (*n* = 90)			Occasional ICGFA users (*n* = 57)			ICGFA non-users (*n* = 53)			Overall *p* value
	Mean	Median	SD	Mean	Median	SD	Mean	Median	SD	
“It reduces anastomotic leak”	4.13	4	0.75	3.46	4	0.97	3.45	4	0.89	2.993 × 10^− 7^
“It is easy to incorporate into clinical practice”	4.63	5	0.53	3.74	4	1.04	3.19	3	0.96	< 2.2 × 10^− 16^

Responses were registered on a Likert scale: 1, strongly disagree; 2, disagree; 3, neutral; 4, agree; 5, strongly agree. Abbreviation: ICGFA, indocyanine green fluorescence angiography; SD, standard deviation

**Table 3:** Results of pair-wise comparisons using wilcoxon rank sum test in table 2

		Non-user	Occasional user
It reduces anastomotic leak	Occasional user	0.98	-
	Routine user	7.8 × 10^− 6^	1.1 × 10^− 5^
It is easy to incorporate into clinical practice	Occasional user	0.0042	-
	Routine user	2.2 × 10^− 16^	2.3 × 10^− 8^

For the statement ‘it is easy to incorporate into routine clinical practice’, 78 (39%) “strongly agreed”, 65 (32.5%) “agreed”, 39 (19.5%) “neither agreed nor disagreed”, 14 (7%) “disagreed”, and 4 (2%) “strongly disagreed”. Routine ICGFA users agreed with this statistically more than occasional ICGFA users (*p* = 2.3 × 10^− 8^) and non-users (*p* = 2.2 × 10^− 16^) (Fig. 1; Tables 2 and 3). Italian surgeons agreed with the statement more than non-Italian surgeons (*p* = 0.038) but there was no difference in opinions between consultants and non-consultants (*p* = 0.17), Irish and non-Irish (*p* = 0.071), and between male and female (*p* = 0.87).

### Factors and challenges affecting adoption

**Fig. 2:**
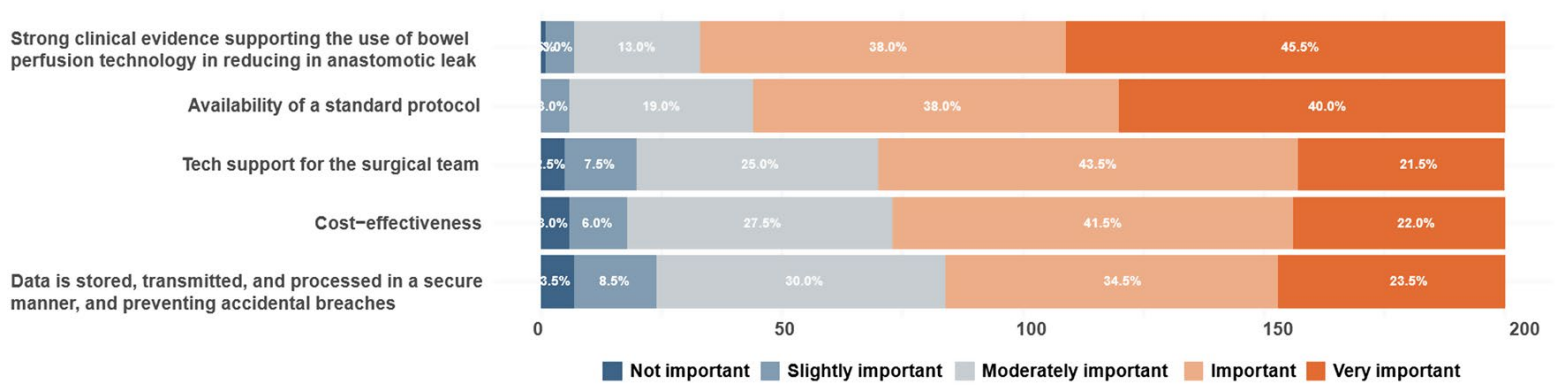
The stacked bar plot shows important factors affecting surgeon’s decision to incorporate colonic perfusion assessment technology into routine clinical practice

“Strong clinical evidence supporting the use of bowel perfusion assessment technology in reducing anastomotic leak” and “availability of a standard protocol” were rated as “important” or “very important” by 83.5% and 78% respectively (see Fig. 2). There was no statistically significant difference in perception regarding factors affecting adoption among surgeons who routinely use ICGFA, those who selectively and rarely use ICGFA, and those who do not use ICGFA at all (Table 4), as well as between consultants and non-consultants and between Irish and non-Irish surgeons (supplementary). There was statistically significant difference in opinions regarding strong clinical evidence (*p* = 0.0406), standard protocol (*p* = 0.0435) and data security (*p* = 0.04006) between Italian and non-Italian surgeons with non-Italian rating it more important than Italian surgeons. There was significant difference regarding strong clinical evidence (*p* = 0.0388), standard protocol (*p* = 0.04212), and tech support (*p* = 0.02836) between male and female, with female surgeons rating these as more important.

**Table 4:** Important factors influencing decision in implementing intraoperative colonic perfusion assessment technology routinely, as perceived by surgeons who routinely use ICGFA, those who selectively and rarely use ICGFA, and those who do not use ICGFA at all

	Routine ICGFA users (*n* = 90)			Occasional ICGFA users (*n* = 57)			ICGFA non-users (*n* = 53)			Overall *p* value
	Mean	Median	SD	Mean	Median	SD	Mean	Medium	SD	
Strong clinical evidence supporting the use of bowel perfusion assessment technology in reducing anastomotic leak	4.34	4.5	0.79	4.19	4	0.85	4.15	4	0.86	0.3277
Availability of a standard protocol	4.19	4	0.81	4.12	4	0.89	4.11	4	0.82	0.8632
Tech support for the surgical team	3.66	4	1.05	3.67	4	0.95	3.96	4	0.78	0.2038
Cost-effectiveness	3.82	4	0.94	3.60	4	1.13	3.74	4	0.81	0.5615
Data is stored, transmitted, and processed in a secure manner, and preventing accidental breaches	3.71	4	0.99	3.56	3	1.18	3.68	4	0.96	0.751

Responses were registered on a Likert scale: 1, not important; 2, slightly important; 3, moderately important; 4, important; 5, very important. Abbreviation: ICGFA, indocyanine green fluorescence angiography; SD, standard deviation

**Fig. 3:**
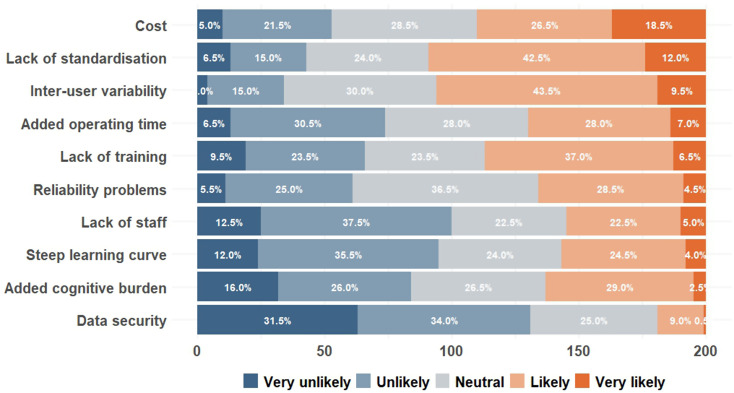
Challenges that are likely to occur when trying to integrate ICGFA technology routinely

Overall, at least 50% of surgeons rated lack of standardisation and inter-user variability as “likely” or “very likely” to present as challenges, while at least 50% rated data security and lack of staff as “unlikely” or “very unlikely” (see Fig. 3). Lack of training and staff, added operating time and cost, reliability problems and data security were perceived differently among surgeons who routinely use ICGFA, those who selectively and rarely use ICGFA, and those who do not use ICGFA at all (see Tables 5 and 6). Added operating time and cost were perceived as challenges more by those who occasionally use ICGFA compared to those who routinely use ICGFA (see Tables 5 and 6). Consultants and non-consultants perceived all factors similarly with no significant differences, except inter-user variability (*p* = 0.04257). Added cognitive burden (*p* = 0.000637) and steep learning curve (*p* = 0.000212) were perceived as challenges more by Irish surgeons compared to non-Irish surgeons. Italian surgeons perceived added cognitive burden as significant barriers compared to non-Italian surgeons (*p* = 0.041), whereas non-Italian surgeons perceived cost and inter-user variability as significant barriers compared to Italian surgeons (*p* = 0.0009 and *p* = 0.0459, respectively). Female surgeons perceived added operating time (*p* = 0.01432) as significant challenges compared to male surgeons. In Ireland, cost, lack of standardisation, inter-user variability and lack of training were most cited barriers. In Italy, lack of standardization, added cognitive burden and inter-user variability were the three most cited barriers.

**Table 5:** Barriers to routinely use intraoperative colonic perfusion assessment technology perceived by surgeons who routinely use ICGFA, those who occasionally use ICGFA, and those who do not use ICGFA at all

	Routine ICGFA users (*n* = 90)			Occasional ICGFA users (*n* = 57)			ICGFA non-users (*n* = 53)			Overall *p* value
	Mean	Median	SD	Mean	Median	SD	Mean	Median	SD	
Added cognitive burden	2.86	3	1.08	2.56	2.5	1.06	2.92	3	1.15	0.203
Lack of training	2.93	3	1.12	2.86	3	1.08	3.55	4	1.02	0.00158
Steep learning curve	2.57	2	1.15	2.75	3	1.06	2.98	3	0.95	0.07351
Lack of staff	2.42	2	1.04	2.67	3	1.14	3.21	3	1.01	0.0001615
Lack of standardisation	3.29	4	1.15	3.40	4	1.10	3.53	4	0.93	0.5823
Added operating time	2.59	2.5	0.89	3.12	3	1.10	3.51	4	1.05	3.209 × 10^− 6^
Cost/lack of funding	2.93	3	1.05	3.61	4	1.16	3.66	4	1.13	8.226 × 10^− 5^
Inter-user variability	3.4	4	0.98	3.44	3	0.93	3.49	4	0.85	0.908
Reliability problems	2.89	3	0.95	2.93	3	1.02	3.32	3	0.89	0.01326
Data security and patient privacy concerns	1.94	2	0.94	2.07	2	0.98	2.51	2	0.95	0.003072

Responses were registered on a Likert scale: 1, very unlikely; 2, unlikely; 3, neutral; 4, likely; 5, very likely. Abbreviation: ICGFA, indocyanine green fluorescence angiography; SD, standard deviation

**Table 6:** Results of pair-wise comparisons using wilcoxon rank sum test in table 5. Only significant barriers (*p* < 0.05) are included in this table

		Non-user	Occasional user
Lack of training	Occasional user	0.0026	-
	Routine user	0.0026	0.7115
Lack of staff	Occasional user	0.017	-
	Routine user	7.7 × 10^− 5^	0.186
Added operating time	Occasional user	0.0742	-
	Routine user	4.1 × 10^− 6^	0.0031
Cost/lack of funding	Occasional user	0.84293	-
	Routine user	0.00049	0.00091
Reliability problems	Occasional user	0.038	-
	Routine user	0.013	0.785
Data security and patient privacy concerns	Occasional user	0.0372	-
	Routine user	0.0019	0.4309

### Level of clinical and cost-benefit required

**Fig. 4:**
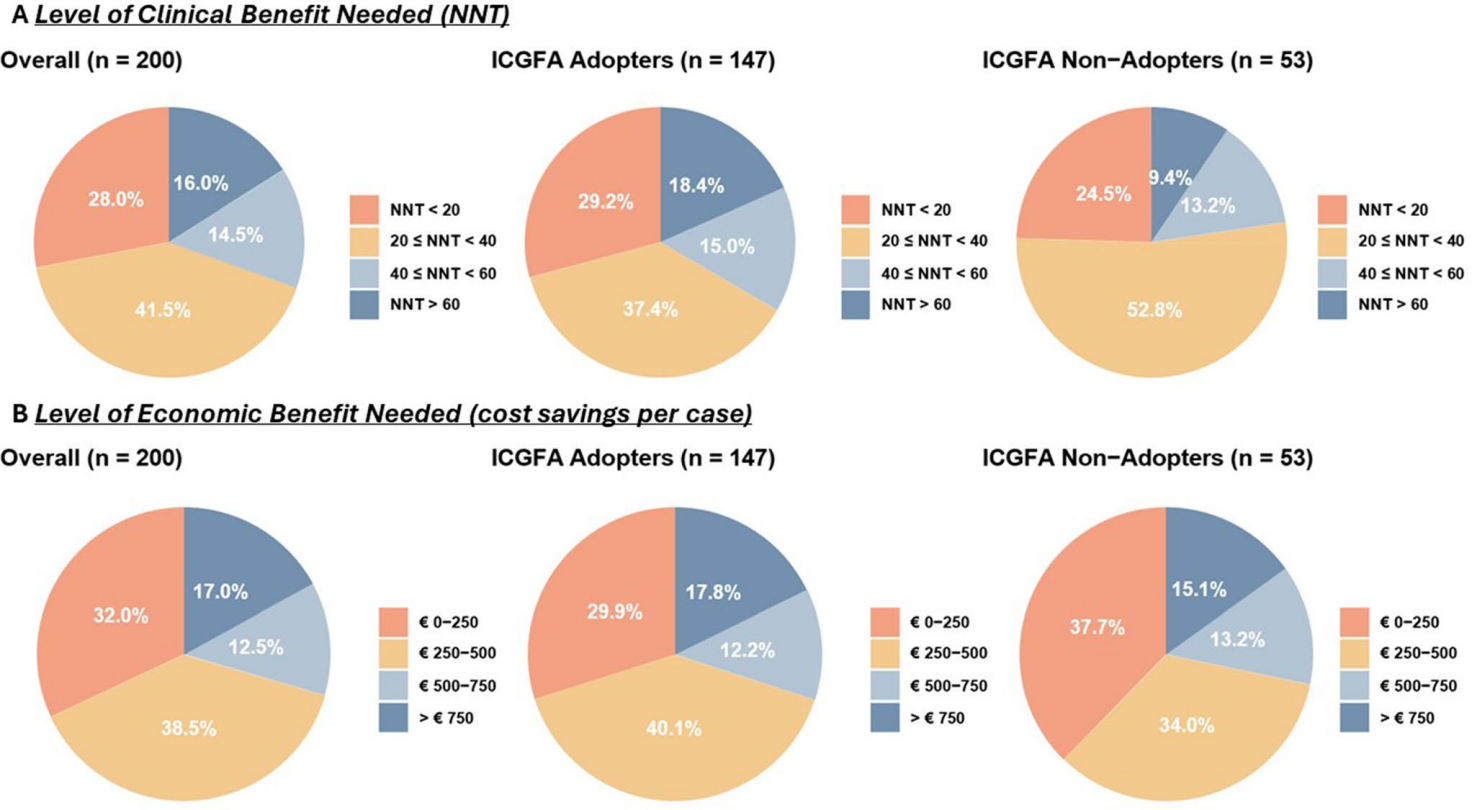
Level of clinical (number needed to treat (NNT)) and economic benefits (cost savings per case) needed to justify the routine use of ICGFA

Most surgeons (41.5%) believe that a number needed to treat between 20 and 40 is acceptable for routinely using ICGFA to reduce leak rates, regardless of whether they currently use the technology or not (see Fig. 4). Overall, most surgeons (38.5%) believe cost savings between €250–500 per case reasonable to support routine use, while 32% stated €0-250 acceptable with 17% considering savings over €750 needed. Similar trends were seen in Ireland and Italy regarding a NNT between 20 and 40 (43.2% and 35.1% respectively) and acceptable cost savings €250–500 (36.4% and 42.1% respectively).

## Discussion

With increasing clinical trial evidence, including now large randomised controlled trials individually and in aggregation, we analysed perceptions and considerations regarding integrating a colonic perfusion assessment technology into routine clinical practice. As ICGFA is the primary technology available and implemented generally, and certainly among respondents here, impressions regarding the level of clinical and cost-effectiveness needed for its routine adoption were specifically explored. The respondents comprise a high degree of ICGFA and indeed bowel perfusion technology adopters and express generally confident and positive views regarding this additive step for colorectal operations. Indeed, in comparison to a previous similar study in a single country [with 44.8% of Italian survey respondents expressing agreement regarding ICGFA and leak reduction, 18], confidence seems to be increasing. Notably however some respondents reject the usefulness of ICGFA regarding AL. Surgeons who use ICGFA selectively, in high-risk cases, and rarely do not strongly agree with the benefits of ICGFA compared to those who use it routinely in all cases.

In this predominantly European survey, lack of standardisation and inter-user variability were most often cited as likely challenges when integrating a bowel perfusion technology into routine clinical practice. Interestingly, surgeons felt that the added cognitive burden when using ICGFA, data security (including video recording), and steep learning curve were unlikely to present challenges, which differs from a previous North American survey where lack of confidence in current evidence, lack of training and lack of awareness were the most cited barriers to using ICGFA [19]. This may be because evidence has strengthened even in the time since the prior survey [7]. After all, recent studies have been completed outside of the USA. Two additional major RCTs (IntAct and ICG-COLORAL) are due to report this year [20, 21]. With the forthcoming results of these major RCTs and the results of the EssentiAL trial and AVOID [8, 9], attention should shift to developing standardised protocols and guidelines as we transition toward making it the standard care.

Regarding NNT and cost-benefit impact, recent evidence reflects an NNT for ICGFA of 22 and a cost benefit of €2,664 per case when ICGFA is used for vascularisation [7, 22]. Vettoretto et al. and the Italian Society of Endoscopic Surgery health technology assessment report revealed the difference in the average economic value of the patient’s pathway when ICGFA was used compared to when it was not used in colorectal surgery was €1688.08 per patient and additionally a budget impact analysis revealed a reduction in costs with a potential 16% decrease in budget with ICGFA use [22]. In Canada, the cost benefit is CAD $192.22 per case with ICGFA use [10].

Regarding the learning curve, standardisation and inter-user variability concerns, training pathways, and more broad educational initiatives seem to be needed. Conferences, webinars and potential fluorescence video libraries could all help bring knowledge and confidence to new users. For instance, the International Society for Fluorescence Guided Surgery (ISFGS) provides webinar series and training for surgeons (available at https://isfgs.org/). This could help offset inter-user variability, although it is likely that this, like in any imaging technology, persists even with these efforts [23]. Interestingly, early attempts at standardising acquisition techniques (including camera setup, ICG dose etc.) have not improved interpretation consistency [24]. For these reasons, quantification of the near infra-red fluorescence signal is being increasingly explored [25–29] and a recent study [30] found that the quantitative ICGFA measurements correlate with current subjective methods. Naturally it’s essential that high quality imaging systems are widely available for the technology to find broad application as low-quality imagers can impair the visualisation of dye distribution and make it difficult to obtain an accurate assessment of perfusion. Independent testing has shown variability in equipment performance that can impact the consistency and reliability of results across different surgical centres and even different theatres within the same centre [31]. While computational representation of actionable information (rather than just more data) could really help advance the field and recent breakthroughs in AI applications in this field hold great promise as a solution here too [32], clearly accuracy in scene visualisation is fundamental.

This study has some limitations of course. Even though the survey content was validated by experts in ICGFA, it was not validated quantitatively using the Lawshe scale [33, 34] and by external ICGFA experts. The study may also naturally be limited by response bias, so the findings may not reflect the opinions of all colorectal surgeons, in particular, the survey seems clearly to have attracted those interested in intraoperative colonic perfusion assessment technologies (selection bias). However, only 61.2% of respondents routinely use the technology versus 38.8% who don’t routinely use it (including approximately 8% who use ICGFA rarely) meaning the work still sheds important insights in reflecting concerns still undermining more widespread adoption efforts.

## Conclusion

Most surgeons responding to this survey have considered ICGFA and many trust it reduces anastomotic leaks after colorectal resections. Alongside strong clinical evidence, standardised protocols and methods to streamline its use and interpretation seem needed for it to become standard care. The level of benefit expected seems in line with data already in the literature encouraging next step integration into practice although additional effort is needed to engage, perhaps the majority of, surgeons who have yet to fully engage with the field to understand better their concerns and obstacles.

## Electronic supplementary material

Below is the link to the electronic supplementary material.

Supplementary Material 1

## Data Availability

No datasets were generated or analysed during the current study.
