# Supplementary material for: Colorectal surgeons’ perspectives on the efficacy of intraoperative bowel perfusion technology with a focus on indocyanine green fluorescence angiography
Source: Langenbecks Arch Surg. 2025 Feb 17;410(1):73. doi: 10.1007/s00423-025-03640-9 (PMC11832565; doi:10.1007/s00423-025-03640-9)
Supplement: Supplementary file 1 — Supplementary Material 1 [file 423_2025_3640_MOESM1_ESM.docx]

**Colorectal surgeons’ perceptions on the efficacy of intraoperative bowel perfusion technology with a focus on indocyanine green fluorescence angiography**

Ashokkumar Singaravelu^1^, Philip Mc Entee^1,2^, Patrick A. Boland^1,2^, Alice Moynihan^1,2^, Cathleen McCarrick^1,2^, Alexander L. Vahrmeijer^3^, Alberto Arezzo^4^, Luigi Boni^5^, Roel Hompes^6^, Ronan A. Cahill^1,2^

^1^UCD Centre for Precision Surgery, University College Dublin, Ireland.

^2^Department of Surgery, Mater Misericordiae University Hospital, Dublin, Ireland.

^3^Department of Surgery, Leiden University Medical Center, Leiden, The Netherlands.

^4^Department of Surgical Sciences, University of Turin, Italy.

^5^Department of General and Minimally Invasive Surgery, Fondazione IRCCS Ca’ Granda Ospedale Maggiore Policlinico di Milano, Italy.

^6^Department of Surgery, Amsterdam University Medical Centre, Amsterdam, The Netherlands.

**Corresponding author.**

Prof Ronan A. Cahill, 47 Eccles Street, Dublin 7, Ireland.

Email: [ronan.cahill@ucd.ie](mailto:ronan.cahill@ucd.ie) Telephone: 00353 1 716 4597 ORCID ID: 0000-0002-1270-4000

**Supplementary Materials - Index**

| **Supplementary Figures and Tables** |  |
| --- | --- |
| Figure S1 | *page. 2* |
| Figure S2 | *page. 2* |
| Figure S3 | *page. 3* |
| Figure S4 | *page. 3* |
| Table S1 | *page. 4* |
| Table S2 | *page. 5* |
| Table S3 | *page. 5* |
| Table S4 | *page. 6* |
| Table S5 | *page. 7* |
| Table S6 | *page. 8* |
| Table S7 | *page. 9* |
| Table S8 | *page. 10* |
| Table S9 | *page. 11* |
| **Survey Questionnaire** | *page. 12* |
|  |  |

**Results**


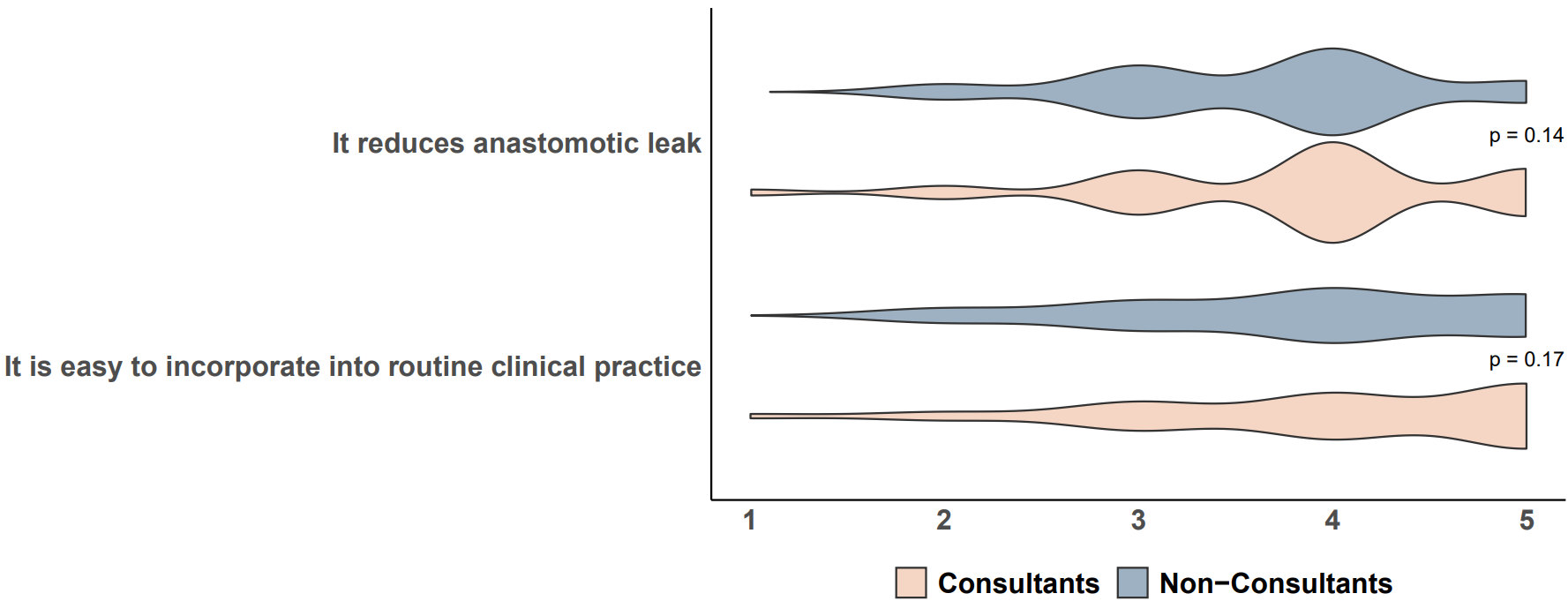


**Figure S1.** Violin plot showing the distribution of surgeons’ opinions on each statement, stratified by level of training. Responses were registered on a five-point Likert scale ranging from 1 (strongly disagree), 2 (disagree), 3 (neutral), 4 (agree), to 5 (strongly agree).


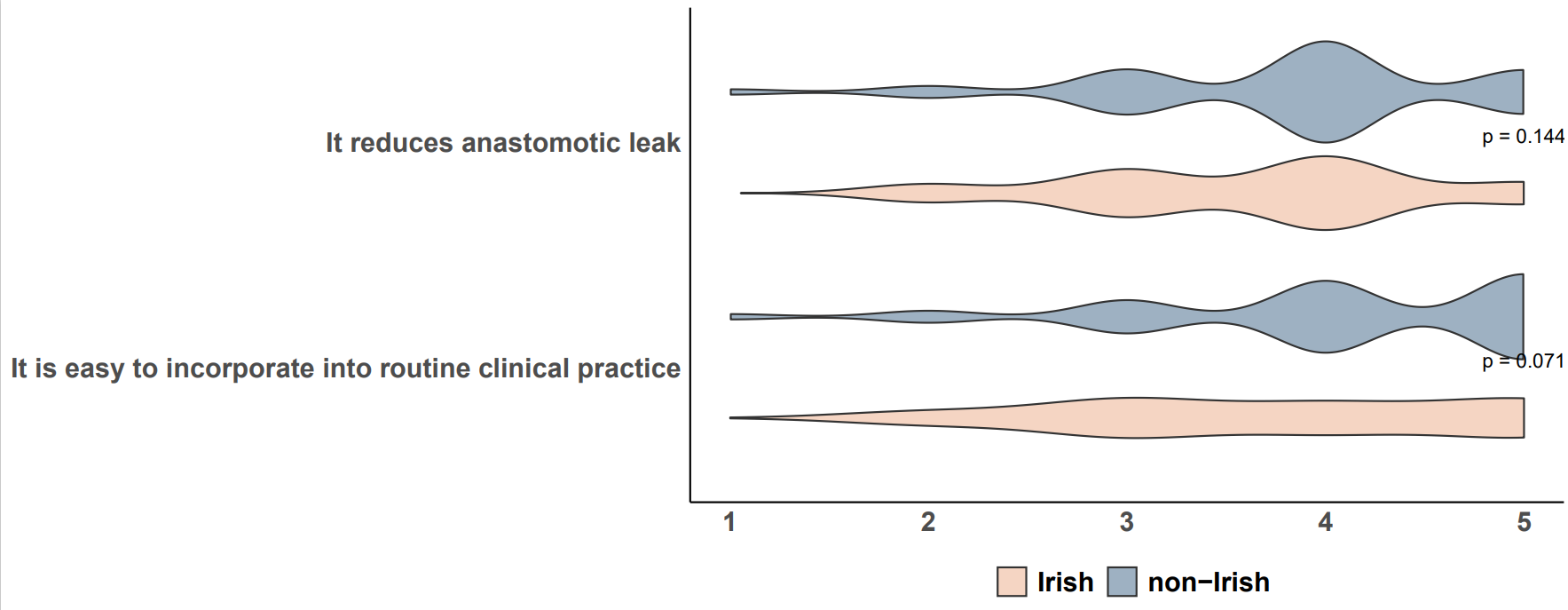


**Figure S2.** Violin plot showing the distribution of opinions of Irish and non-Irish surgeons on each statement regarding bowel perfusion technology. Responses were registered on a five-point Likert Scale ranging from 1 (strongly disagree), 2 (disagree), 3 (neutral), 4 (agree), to 5 (strongly agree).


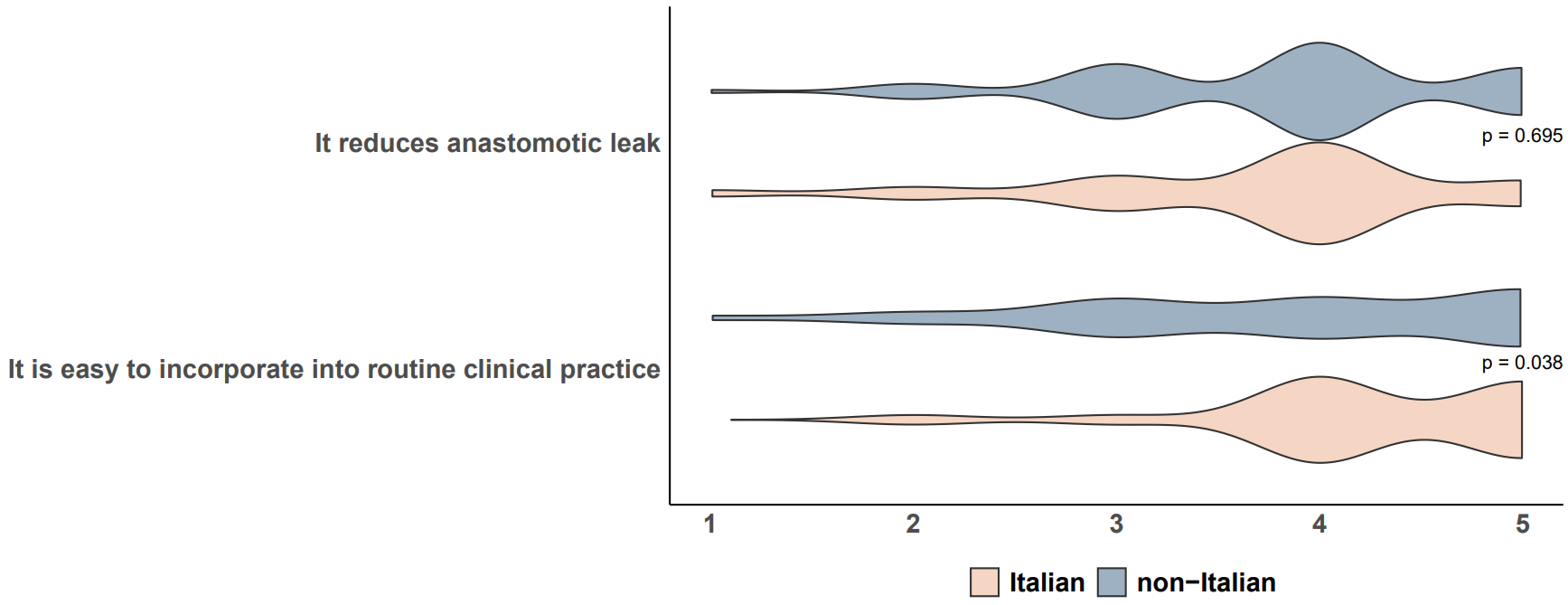


**Figure S3.** Violin plot showing the distribution of opinions of Italian and non-Italian surgeons on each statement regarding bowel perfusion technology. Responses were registered on a five-point Likert scale ranging from 1 (strongly disagree), 2 (disagree), 3 (neutral), 4 (agree), to 5 (strongly agree).
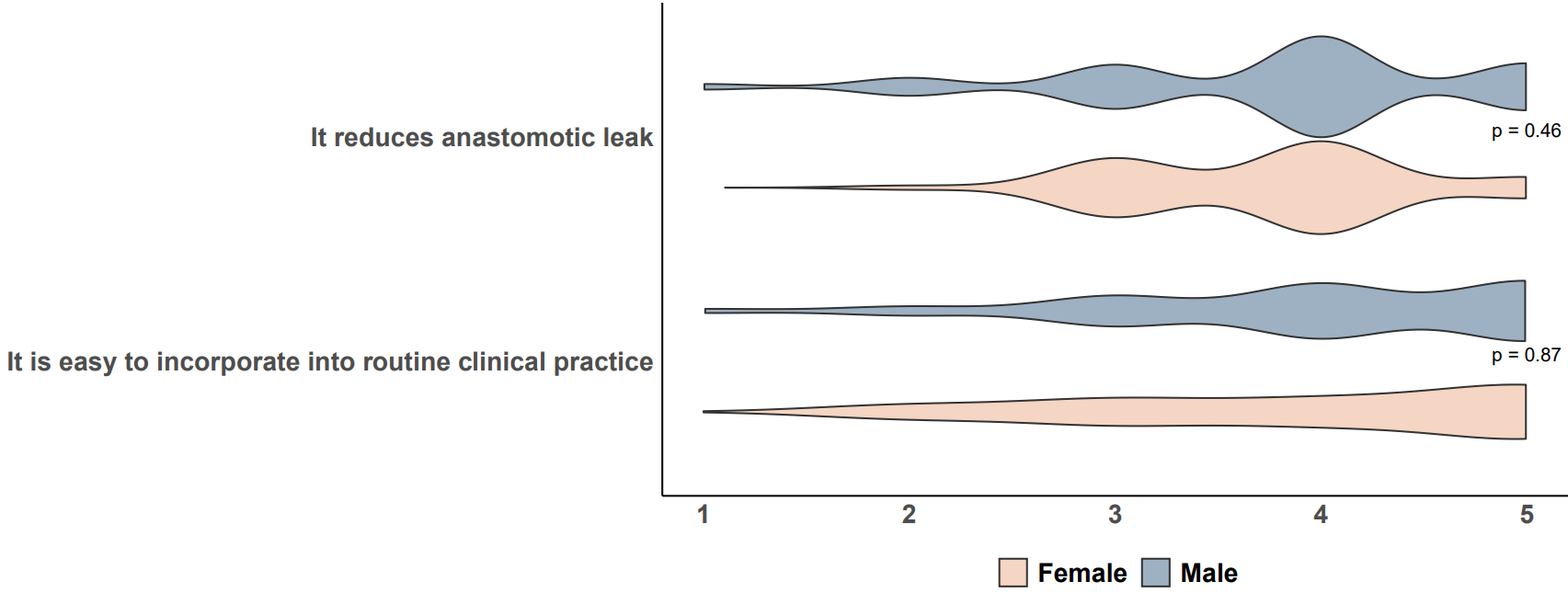


**Figure S4.** Violin plot showing the distribution of surgeon’s opinions based on gender. Responses were registered on a five-point Likert scale ranging from 1 (strongly disagree), 2 (disagree), 3 (neutral), 4 (agree), to 5 (strongly agree).

**Table S1.** Geographical information of participants**.**

| **Country** | **No. (%)** |
| --- | --- |
| Australia | 1 (0.50%) |
| Austria | 2 (1.00%) |
| Bosnia and Herzegovina | 2 (1.00%) |
| Bulgaria | 5 (2.50%) |
| Croatia | 1 (0.50%) |
| Czech Republic | 2 (1.00%) |
| Denmark | 1 (0.50%) |
| France | 4 (2.00%) |
| Germany | 7 (3.50%) |
| Greece | 10 (5.00%) |
| India | 5 (2.50%) |
| Iraq | 1 (0.50%) |
| Ireland | 44 (22.00%) |
| Italy | 57 (28.50%) |
| Japan | 2 (1.00%) |
| Latvia | 1 (0.50%) |
| Lithuania | 2 (1.00%) |
| Luxembourg | 1 (0.50%) |
| Malta | 1 (0.50%) |
| Mexico | 2 (1.00%) |
| The Netherlands | 8 (4.00%) |
| Nigeria | 1 (0.50%) |
| Peru | 1 (0.50%) |
| Poland | 2 (1.00%) |
| Portugal | 3 (1.00%) |
| Romania | 5 (2.50%) |
| Serbia | 3 (1.50%) |
| Slovakia | 1 (0.50%) |
| Slovenia | 1 (0.50%) |
| Spain | 4 (2.00%) |
| Sweden | 2 (1.00%) |
| Switzerland | 3 (1.50%) |
| Turkey | 6 (3.00%) |
| Ukraine | 2 (1.00%) |
| United Kingdom | 6 (3.00%) |
| Vietnam | 1 (0.50%) |
| **Total** | **200** |

**Table S2.** Important factors influencing decision in implementing intraoperative colonic perfusion assessment technology routinely, as perceived by consultants and non-consultants.

|  | **Consultants (n=143)** | | | **Non-consultants (n=57)** | | | **p value** |
| --- | --- | --- | --- | --- | --- | --- | --- |
|  | **Mean** | **Median** | **SD** | **Mean** | **Median** | **SD** |  |
| Strong clinical evidence supporting the use of bowel perfusion assessment technology in reducing anastomotic leak | 4.21 | 4 | 0.80 | 4.35 | 5 | 0.89 | 0.1219 |
| Availability of a standard protocol | 4.15 | 4 | 0.86 | 4.16 | 4 | 0.74 | 0.8678 |
| Tech support for the surgical team | 3.67 | 4 | 0.99 | 3.91 | 4 | 0.86 | 0.09446 |
| Cost-effectiveness | 3.73 | 4 | 0.94 | 3.75 | 4 | 1.03 | 0.688 |
| Data is stored, transmitted, and processed in a secure manner, and preventing accidental breaches | 3.78 | 4 | 1.02 | 3.59 | 4 | 1.06 | 0.5722 |

*Responses were registered on a Likert scale: 1, not important; 2, slightly important; 3, moderately important; 4, important; 5, very important. Abbreviation. SD, standard deviation.*

**Table S3.** Important factors influencing decision in implementing intraoperative colonic perfusion assessment technology routinely, as perceived by Irish and non-Irish surgeons.

|  | **Irish (n=44)** | | | **Non-Irish (n=156)** | | | **p value** |
| --- | --- | --- | --- | --- | --- | --- | --- |
|  | **Mean** | **Median** | **SD** | **Mean** | **Median** | **SD** |  |
| Strong clinical evidence supporting the use of bowel perfusion assessment technology in reducing anastomotic leak | 4.43 | 5 | 0.65 | 4.19 | 4 | 0.87 | 0.159 |
| Availability of a standard protocol | 4.11 | 4 | 0.86 | 4.16 | 4 | 0.82 | 0.802 |
| Tech support for the surgical team | 3.70 | 4 | 0.92 | 3.75 | 4 | 0.97 | 0.645 |
| Cost-effectiveness | 3.64 | 4 | 0.96 | 3.76 | 4 | 0.97 | 0.397 |
| Data is stored, transmitted, and processed in a secure manner, and preventing accidental breaches | 3.55 | 4 | 1.05 | 3.69 | 4 | 1.03 | 0.4008 |

*Responses were registered on a Likert scale: 1, not important; 2, slightly important; 3, moderately important; 4, important; 5, very important. Abbreviation. SD, standard deviation.*

**Table S4.** Important factors influencing decision in implementing intraoperative colonic perfusion assessment technology routinely, as perceived by Italian and non-Italian surgeons.

|  | **Italian (n=57)** | | | **Non-Italian (n=143)** | | | **p value** |
| --- | --- | --- | --- | --- | --- | --- | --- |
|  | **Mean** | **Median** | **SD** | **Mean** | **Median** | **SD** |  |
| Strong clinical evidence supporting the use of bowel perfusion assessment technology in reducing anastomotic leak | 4.05 | 4 | 0.91 | 4.33 | 4 | 0.78 | 0.0406 |
| Availability of a standard protocol | 3.98 | 4 | 0.78 | 4.22 | 4 | 0.84 | 0.0435 |
| Tech support for the surgical team | 3.65 | 4 | 0.96 | 3.78 | 4 | 0.96 | 0.3867 |
| Cost-effectiveness | 3.56 | 4 | 0.97 | 3.80 | 4 | 0.96 | 0.1167 |
| Data is stored, transmitted, and processed in a secure manner, and preventing accidental breaches | 3.42 | 3 | 1.01 | 3.76 | 4 | 1.03 | 0.04006 |

*Responses were registered on a Likert scale: 1, not important; 2, slightly important; 3, moderately important; 4, important; 5, very important. Abbreviation. SD, standard deviation.*

**Table S5.** Important factors influencing decision in implementing intraoperative colonic perfusion assessment technology routinely, as perceived by male and female surgeons.

|  | **Male (n=156)** | | | **Female (n=42)** | | | **p value** |
| --- | --- | --- | --- | --- | --- | --- | --- |
|  | **Mean** | **Median** | **SD** | **Mean** | **Median** | **SD** |  |
| Strong clinical evidence supporting the use | 4.20 | 4 | 0.81 | 4.43 | 5 | 0.88 | 0.0388 |
| Availability of a standard protocol | 4.09 | 4 | 0.84 | 4.38 | 5 | 0.75 | 0.04212 |
| Tech support for the surgical team | 3.65 | 4 | 0.99 | 4.02 | 4 | 0.77 | 0.02836 |
| Cost-effectiveness | 3.71 | 4 | 0.97 | 3.78 | 4 | 0.94 | 0.7066 |
| Data is stored, transmitted, and processed in a secure manner, and preventing accidental breaches | 3.58 | 4 | 1.06 | 3.93 | 4 | 0.88 | 0.07344 |

*Responses were registered on a Likert scale: 1, not important; 2, slightly important; 3, moderately important; 4, important; 5, very important. Abbreviation. SD, standard deviation.*

**Table S6.** Barriers to use intraoperative colonic perfusion assessment technology routinely perceived by consultants and non-consultants.

|  | **Consultants (n=143)** | | | **Non-consultants (n=57)** | | | **p value** |
| --- | --- | --- | --- | --- | --- | --- | --- |
|  | **Mean** | **Median** | **SD** | **Mean** | **Median** | **SD** |  |
| Added cognitive burden | 2.79 | 3 | 1.14 | 2.68 | 3 | 1.03 | 0.4868 |
| Lack of training | 3.03 | 3 | 1.09 | 3.17 | 3 | 1.16 | 0.4227 |
| Steep learning curve | 2.79 | 3 | 1.13 | 2.56 | 2 | 0.92 | 0.2024 |
| Lack of staff | 2.64 | 2 | 1.12 | 2.84 | 3 | 1.04 | 0.2003 |
| Lack of standardisation | 3.32 | 3 | 1.10 | 3.54 | 4 | 1.00 | 0.1404 |
| Added operating time | 2.99 | 3 | 1.07 | 2.98 | 3 | 1.03 | 0.9541 |
| Cost | 3.35 | 3 | 1.15 | 3.25 | 3 | 1.13 | 0.561 |
| Inter-user variability | 3.36 | 3 | 0.88 | 3.61 | 4 | 1.00 | 0.04257 |
| Reliability problems | 2.97 | 3 | 0.95 | 3.12 | 3 | 0.99 | 0.2874 |
| Data security | 2.19 | 2 | 0.95 | 1.96 | 2 | 1.03 | 0.07099 |

*Responses were registered on a Likert scale: 1, very unlikely; 2, unlikely; 3, neutral; 4, likely; 5, very likely. Abbreviation. SD, standard deviation.*

**Table S7.** Barriers to use intraoperative colonic perfusion assessment technology routinely perceived by Irish and non-Irish surgeons.

|  | **Irish (n=44)** | | | **Non-Irish (n=156)** | | | **p value** |
| --- | --- | --- | --- | --- | --- | --- | --- |
|  | **Mean** | **Median** | **SD** | **Mean** | **Median** | **SD** |  |
| Added cognitive burden | 2.25 | 2 | 1.05 | 2.90 | 3 | 1.08 | 0.000637 |
| Lack of training | 3.14 | 3 | 1.19 | 3.06 | 3 | 1.08 | 0.6639 |
| Steep learning curve | 2.20 | 2 | 0.69 | 2.88 | 3 | 1.12 | 0.000212 |
| Lack of staff | 2.68 | 2 | 0.97 | 2.71 | 3 | 1.13 | 0.9584 |
| Lack of standardisation | 3.48 | 4 | 0.99 | 3.36 | 4 | 1.10 | 0.5841 |
| Added operating time | 3.14 | 3 | 0.92 | 2.94 | 3 | 1.09 | 0.2335 |
| Cost | 3.43 | 4 | 1.12 | 3.29 | 3 | 1.15 | 0.4677 |
| Inter-user variability | 3.34 | 4 | 1.02 | 3.46 | 4 | 0.89 | 0.6064 |
| Reliability problems | 2.84 | 3 | 1.04 | 3.06 | 3 | 0.94 | 0.1601 |
| Data security | 1.95 | 2 | 0.85 | 2.19 | 2 | 1.03 | 0.1767 |

*Responses were registered on a Likert scale: 1, very unlikely; 2, unlikely; 3, neutral; 4, likely; 5, very likely. Abbreviation. SD, standard deviation.*

**Table S8.** Barriers to use intraoperative colonic perfusion assessment technology routinely perceived by Italian and non-Italian surgeons.

|  | **Italian (n=57)** | | | **Non-Italian (n=143)** | | | **p value** |
| --- | --- | --- | --- | --- | --- | --- | --- |
|  | **Mean** | **Median** | **SD** | **Mean** | **Median** | **SD** |  |
| Added cognitive burden | 3.02 | 3 | 1.11 | 2.66 | 3 | 1.09 | 0.041017 |
| Lack of training | 2.98 | 3 | 1.13 | 3.11 | 3 | 1.10 | 0.4334 |
| Steep learning curve | 2.77 | 3 | 1.14 | 2.71 | 3 | 1.06 | 0.6999 |
| Lack of staff | 2.53 | 2 | 1.09 | 2.77 | 3 | 1.09 | 0.1835 |
| Lack of standardisation | 3.17 | 3 | 1.20 | 3.47 | 4 | 1.02 | 0.1427 |
| Added operating time | 2.75 | 3 | 1.09 | 3.08 | 3 | 1.03 | 0.05489 |
| Cost | 2.91 | 3 | 1.01 | 3.48 | 4 | 1.16 | 0.0009188 |
| Inter-user variability | 3.23 | 3 | 0.96 | 3.52 | 4 | 0.89 | 0.0459 |
| Reliability problems | 2.93 | 3 | 0.88 | 3.05 | 3 | 0.99 | 0.3852 |
| Data security | 2.19 | 2 | 1.11 | 2.12 | 2 | 0.94 | 0.8286 |

*Responses were registered on a Likert scale: 1, very unlikely; 2, unlikely; 3, neutral; 4, likely; 5, very likely. Abbreviation. SD, standard deviation.*

**Table S9.** Barriers to use intraoperative colonic perfusion assessment technology routinely perceived by male and female surgeons.

|  | **Male (n=156)** | | | **Female (n=44)** | | | **p value** |
| --- | --- | --- | --- | --- | --- | --- | --- |
|  | **Mean** | **Median** | **SD** | **Mean** | **Median** | **SD** |  |
| **Added cognitive burden** | 2.76 | 3 | 1.15 | 2.79 | 3 | 1.10 | 0.9151 |
| **Lack of training** | 3.07 | 3 | 1.10 | 3.12 | 3.5 | 1.16 | 0.7329 |
| **Steep learning curve** | 2.69 | 3 | 1.04 | 2.93 | 3 | 1.22 | 0.2839 |
| **Lack of staff** | 2.69 | 2 | 1.09 | 2.79 | 3 | 1.10 | 0.6309 |
| **Lack of standardisation** | 3.38 | 4 | 1.09 | 3.45 | 4 | 0.98 | 0.7664 |
| **Added operating time** | 2.89 | 3 | 1.06 | 3.33 | 3.5 | 1.01 | 0.01432 |
| **Cost** | 3.25 | 3 | 1.17 | 3.59 | 4 | 1.02 | 0.09674 |
| **Inter-user variability** | 3.45 | 4 | 0.92 | 3.43 | 4 | 0.93 | 0.9047 |
| **Reliability problems** | 3.02 | 3 | 0.98 | 3 | 3 | 0.95 | 0.8839 |
| **Data security** | 2.12 | 2 | 0.97 | 2.17 | 2 | 0.99 | 0.8155 |

*Responses were registered on a Likert scale: 1, very unlikely; 2, unlikely; 3, neutral; 4, likely; 5, very likely. Abbreviation. SD, standard deviation.*

**Supplementary File – Survey Questionnaire**

**Information Sheet**

1. Introductory statement

I am Mr. Ashokkumar Singaravelu from the School of Medicine at University College Dublin, and I am doing an intercalated MSc by research degree. I am conducting a research study titled “Survey of perceptions regarding intraoperative bowel perfusion assessments.”

1. What is this research about?

This survey aims to explore the perspectives of colorectal surgeons regarding the utilization of intraoperative bowel perfusion assessment technology. The objectives are (1) to assess current practice, (2) determine perceived benefits, (3) explore challenges when implementing intraoperative bowel perfusion assessment, (4) investigate the factors that influence the adoption of tissue perfusion assessment technology in plastic surgery, (5) assess the perceived cost-effectiveness, and (6) understand the perspectives on the clinical effectiveness and impact on patient outcomes.

1. Why am I doing this research?

The survey is conducted to enhance our understanding of the potential benefits and challenges associated with the use of intraoperative bowel perfusion assessment technology in colorectal surgery.

1. How will your data be used?

The data collected in this study will be used solely for research purposes. It will be analyzed and published in academic journals and/or presented at conferences. All data will be anonymized to protect participants’ identities.

1. What will happen if you decide to take part in this research study?

If you choose to participate, you will be asked to complete a survey questionnaire. The survey is estimated to take approximately 5 minutes to complete, depending on the depth of your responses.

1. How will I protect your privacy?

All information you provide will be kept confidential and stored securely. Your responses will be anonymized. Participants should not include any identifiable information in the open-ended questions.

1. What are the benefits of taking part in this research study?

Study participants will receive no specific benefits. The data will be submitted for publication, and your insights will contribute to our understanding of the current usage of intraoperative bowel perfusion assessment technology and the identification of barriers to its routine implementation in Ireland and the UK.

1. What are the risks of taking part in this research study?

There are no risks to participants in this survey.

1. Can I change my mind at any stage and withdraw from the study?

Yes, you can withdraw from the study at any time.

1. How will I find out what happens with this project?

Only the researchers will have access to the raw data. The results will be submitted for publication.

***Consent form***

For ethical reasons it is important that you give your fully informed consent to participate in this study.

If you would like to participate, please complete this section.

DECLARATION

I have read the information sheet (previous page) and have had time to consider whether to take part in this study. I understand that my participation is voluntary (it is my choice) and that I am free to withdraw from the research at any time without disadvantage or penalty. I confirm that I am over the age of 18. I agree to take part in this research.

I agree that the data arising from this research can be published and that I will not be identified in any way.

If you have any questions about this research, please contact me at [ashokkumar.singaravelu@ucdconnect.ie](mailto:ashokkumar.singaravelu@ucdconnect.ie)

I consent and would like to continue with the survey.

Yes (please continue to the next section and complete the survey)

No (your information will NOT be kept or analyzed. Please close this window)

**Questions**

**Demographic information**

| Position | Options: consultant, registrar, senior house officer, intern |
| --- | --- |
| Gender | Options:   1. Male 2. Female 3. Prefer not to say |
| Surgical experience | Options:   1. 0-5 years into practice 2. 6-10 years into practice 3. 11-15 years into practice 4. 16-20 years into practice 5. >20 years into practice |

**Current use of bowel perfusion assessment in practice**

| List of most frequently used assessment modalities  1. Indocyanine green fluorescence angiography  2. Diffuse reflectance spectroscopy  3. Hyperspectral imaging  4. Laser speckle contrast imaging | |
| --- | --- |
| **(Q)** Do you use any of the above-mentioned tissue perfusion technology intraoperatively? | Options:   1. Yes 2. No |
| If Yes to the above question **Q**, the following questions will be asked.  If No to the above question **Q,** then the survey will move on to next section | |
| Which bowel perfusion assessment technology do you use to assess colorectal anastomoses? (select all that apply) | Options:   1. Indocyanine green fluorescence angiography 2. Diffuse reflectance spectroscopy 3. Hyperspectral imaging 4. Laser speckle contrast imaging |
| Which one of the following bowel perfusion methods do you believe has the greatest impact in reducing the incidence of anastomotic leak? | Options:   1. Indocyanine green fluorescence angiography 2. Diffuse reflectance spectroscopy 3. Hyperspectral imaging 4. Laser speckle contrast imaging |
| How often do you use bowel perfusion assessment technology? (select all that apply) | Options:   1. Routinely in all patients 2. Selectively in patients with high-risk comorbidities (e.g., obesity, smoking, etc) 3. Selectively in complex surgical procedures 4. Rarely |
| Do you use bowel perfusion assessment data in combination with other intraoperative monitoring tools (e.g., blood pressure monitoring, pulse oximetry, ECG or EKG, etc) to make decisions? | Options:   1. Yes 2. No 3. Don’t know |
| Have you done any experimental research on intraoperative bowel perfusion assessment? | Options:   1. Animal study 2. Human study 3. Laboratory study 4. Both animal and human tissues 5. No, I have not done any experimental research |

**Impact on Surgical Decisions**

| How often has intraoperative bowel perfusion assessment influenced your surgical decision-making? | Options:   1. Very often 2. Often 3. Occasionally 4. Rarely 5. Never 6. I don’t use bowel perfusion assessment technology |
| --- | --- |
| How confident are you in the accuracy and reliability of the bowel perfusion assessment methods you use? | Options:   1. Not confident 2. Slightly confident 3. Moderately confident 4. Confident 5. Very confident 6. I don’t use bowel perfusion assessment technology |

**Opportunities**

| Do you agree on the following statements regarding the use of intraoperative bowel perfusion technology?  1 = strongly disagree, 2 = disagree, 3 = neutral, 4 = agree, 5 = strongly agree | |
| --- | --- |
| 1. It has the potential to reduce anastomotic leaks in colorectal surgery | 1 2 3 4 5 |
| 1. It is convenient to use | 1 2 3 4 5 |
| 1. It is easy to incorporate into routine clinical practice | 1 2 3 4 5 |

**Challenges**

| **How likely is it that the following challenges will occur when trying to integrate a bowel perfusion assessment technology into routine clinical practice?**  1 = Very unlikely, 2 = unlikely, 3 = neutral, 4 = likely, 5 = very likely | |
| --- | --- |
| 1. Lack of experience 2. Lack of training 3. Steep learning curve 4. Lack of staff 5. Lack of standardization 6. Additional operating time 7. Cost/lack of funding 8. Observer dependent 9. Reliability problems 10. Data security and patient privacy concerns | 1 2 3 4 5  1 2 3 4 5  1 2 3 4 5  1 2 3 4 5  1 2 3 4 5  1 2 3 4 5  1 2 3 4 5  1 2 3 4 5  1 2 3 4 5  1 2 3 4 5  1 2 3 4 5 |

**Incorporating intraoperative tissue perfusion technology in routine clinical approaches**

| **What would be the main factors affecting your decision to incorporate bowel perfusion assessment technology into routine clinical practice?**  1 = Not important, 2 = Slightly important, 3 = moderately important, 4 = important, 5 = very important | |
| --- | --- |
| 1. Clinical evidence supporting the use of bowel perfusion assessment techniques in reducing anastomotic leak. 2. Cost-effectiveness 3. Tech support for the surgical team 4. Availability of a standard protocol 5. Time-efficiency 6. Data is stored, transmitted, and processed in a secure manner, and preventing accidental breeches | 1 2 3 4 5  1 2 3 4 5  1 2 3 4 5  1 2 3 4 5  1 2 3 4 5  1 2 3 4 5 |
| Should bowel perfusion assessment technology be used routinely in high-risk individuals exclusively, or should it be used in all patients? | Options:   1. High-risk patients only 2. All patients 3. Not sure |

**Cost-benefit of ICG fluorescence imaging**

What do you believe would be a reasonable cost savings per case achieved by implementing routine ICG fluorescence imaging for preventing anastomotic leakage?

| **<€250** | **€250-500** | **€500-750** | **€750-1000** | **>€1000** |
| --- | --- | --- | --- | --- |
|  |  |  |  |  |

**Clinical efficacy of fluorescence imaging**

**What is the maximum acceptable number needed to treat (NNT) value, in your opinion, to justify the routine implementation of ICG fluorescence imaging for preventing anastomotic leaks?**

**Description:** If the number needed to treat (NNT) is N. This means that for every N number of patients, 1 patient will benefit from fluorescence imaging intervention, preventing anastomotic leaks.

**Options:**

1. **Less than 20**
2. **Between 20 and 40**
3. **Between 40 and 60**
4. **More than 60**

*End of survey*
